# An Overview of Current Biomarkers, the Therapeutic Implications, and the Emerging Role of hERG1 Expression in Gastric Cancer: A Literature Review

**DOI:** 10.7759/cureus.47501

**Published:** 2023-10-23

**Authors:** Yahya Abdullah

**Affiliations:** 1 Internal Medicine, Countess of Chester Hospital NHS Foundation Trust, Chester, GBR

**Keywords:** herg1, predictive medicine, targeted therapy, monoclonal antibody, personalised medicine, diagnosis & prognosis, ion channel, molecular biomarker, gastric cancer (gc)

## Abstract

Gastric cancer remains one of the most commonly diagnosed cancers in the world. It carries a high mortality rate, with cases being more prevalent in the developing world, and has been linked to diet and *Helicobacter pylori* infection. It is a highly heterogeneous disease, with most cases being of a sporadic nature. Most patients present at an advanced stage due to the asymptomatic nature of the early stages of the disease. A multidisciplinary approach is often best implemented to help decide how to best manage individual cases. However, the overall clinical outcome and survival of patients with advanced gastric cancer remain poor. Recent therapeutic advancements focus on the identification of molecular biomarkers associated with gastric cancer that have predictive, diagnostic, and prognostic implications. This enables the development of specific targeted therapies that have shown efficacy in numerous trials, either as monotherapy or in combination with standard chemotherapy. Despite this, tumour heterogeneity and treatment resistance are still issues leading to poor survival outcomes. An emerging approach is focusing efforts on the bidirectional crosstalk between tumour cells and the microenvironment through targeting ion channels. A key player in this is human ether-á-go-go-related gene 1 (*hERG1*). This voltage-gated potassium ion channel has been shown to have predictive, diagnostic, and prognostic significance, enabling the stratification of high-risk individuals. In addition, targeting *hERG1* in combination with chemotherapy has been shown to potentiate tumour regression. This comprehensive literature review will aim to consolidate our understanding of current biomarkers in gastric cancer. The relevance of *hERG1* in gastric cancer as a useful novel biomarker and the potential therapeutic implications as targeted therapy will be explored. This offers a new and personalised approach to helping to manage patients with gastric cancer.

## Introduction and background

Gastric cancer remains one of the most common causes of cancer mortality in the world. It is believed to be the third most common cause of cancer deaths and the fifth leading cancer diagnosed worldwide [1]. It was previously thought the mortality rates of gastric cancer were falling worldwide, although recent trends suggest an upward trend [2]. The incidence of gastric cancer is around 1.1 million cases, with around 770,000 deaths, as estimated in 2020 [3]. This is projected to further increase in 2040, with an estimated incidence of 1.8 million cases and 1.3 million deaths [3]. There is a clear geographical distribution of gastric cancer, with a higher prevalence in the developing world. There are a host of established risk factors associated with gastric cancer, which commonly include a high-salt diet, consumption of processed meats, obesity, advanced age, family history, smoking, and *Helicobacter pylori* [4]. *H. pylori* has indeed been shown to cause focal atrophy, metaplasia, dysplasia, and progression to gastric carcinoma [5]. It is our understanding of the pathophysiology of gastric cancer that helps guide advancements in treatment.

There are two main histological variants of gastric cancer, according to the Lauren Criteria [6]. These are well-differentiated intestinal and undifferentiated diffuse-type adenocarcinomas, both of which exhibit different cellular and molecular characteristics. A third variant, known as the indeterminate type, is less often encountered. Most gastric cancers are sporadic and diagnosed at an advanced stage [7]. However, there are established mutations that genetically predispose individuals to tumour risk syndromes (TRS) [8]. The E-cadherin gene encodes for a tumour suppressor glycoprotein named E-cadherin, which has a pivotal role in cell signalling pathways that modulate proliferation, adhesion, invasion, and migration. Downregulation of the wild-type protein disrupts cell signalling, leading to autosomal dominant diffuse gastric cancer that is highly penetrative, invasive, and carries a poor prognosis [8]. In contrast, the carcinogenesis of intestinal-type gastric cancer may be linked to amplified oncogenes [9]. The mainstay of treatment is evaluation with a multidisciplinary team, and accurate staging helps guide the need for radical surgical resection, radiation, neoadjuvant/adjuvant chemotherapy, and palliation [10]. An emerging approach to treating the high heterogeneity seen in gastric cancer is targeted therapy. Recent developments have been able to identify molecular biomarkers in gastric cancer that help to develop individualised targeted therapy and better understand the prognostic implications [6]. Biomarkers are biological molecules that can be detected in the body and indicate normal or abnormal processes, such as cancer [11]. In cancer, they offer potential implications for predictive, diagnostic, and prognostic outcomes in conjunction with histopathology. The use of biomarkers can help mitigate the standard cytotoxic effects of chemotherapy by instead offering effective stratification of patients and a more personalised treatment approach based on tumour profiling.

A biomarker-based treatment strategy can be seen with human epidermal growth factor receptor-2 (HER2) and trastuzumab. HER2 is a proto-oncogene, and in gastric cancer, it is upregulated [12]. The identification of HER2 is through immunohistochemistry and, if required, in situ hybridisation [13]. Trastuzumab, a monoclonal antibody, is a novel approach that selectively binds to the external domain of the HER2 receptor, causing downregulation of HER2 expression and inhibiting tumour growth [14]. However, tumour resistance may be encountered due to tumour heterogeneity, leading to poor outcomes. This has led to research exploring new areas of neoplastic progression in order to help identify new molecular markers and potential targets for therapy. One promising area is targeting ion channels involved in carcinogenesis. Alterations in ion channels promote cell proliferation, invasiveness, angiogenesis, metastasis, and apoptosis [15]. An example of this is the human ether-á-go-go-related gene 1 (hERG1). This voltage-dependent potassium ion channel is an integral player in neoplastic progression, with pleiotropic effects in the regulation of cell proliferation, tumour cell invasiveness, and neoangiogenesis [16]. The identification of hERG1 as a novel unconventional biomarker through immunohistochemistry offers further supplementation in the diagnosis, prognosis, and treatment of gastric cancer. This comprehensive literature review will aim to provide an overview of the present molecular biomarkers in gastric cancer and associated targeted therapies. In addition, it will synthesise the current findings regarding the significance of hERG1 expression as a biomarker in gastric cancer. Lastly, the potential therapeutic applications of hERG1 will be explored in the context of targeted therapy to help optimise treatment through personalisation.

## Review

Overview of current biomarkers and targeted therapy

There are several biomarkers that are associated with gastric cancer. An example is the human epidermal growth factor receptor-2 (HER2). HER2 is a receptor tyrosine kinase, and the main transduction pathway of HER2 is mediated through ligand-independent heterodimerisation with another family member or self-homodimerisation [17]. This results in the auto-transphosphorylation of tyrosine kinase domains. This initiates a cascade of downstream signalling pathways and transcription of different genes involved in cell proliferation, differentiation, angiogenesis, invasion, and survival [17]. The use of polyclonal and monoclonal antibodies to target the epitope of overexpressed HER2 is best determined by immunohistochemistry, which helps to determine the number of HER2 receptors [18,19]. Conversely, fluorescent in situ hybridisation (FISH) or, more recently, silver-enhanced in situ hybridisation (SISH) can help to evaluate gene amplification by measuring the number of HER2 gene copies [18,19]. HER2 positivity is defined as immunohistochemistry 3+, 2+/FISH+, and 2+/SISH+, which is quantified according to the visibility of membrane staining associated with the degree of magnification [13]. This scoring criterion helps to guide the applicability of anti-HER2-directed therapy [13]. The genetic aberrations associated with overexpression and amplification of HER2 are more frequently seen in the intestinal type than in the diffuse form of gastric cancer [12]. To further this end, the rate of HER2 expression was predominately evident in male patients and macroscopically seen with gastric tumours that were ulcerated (type III) and flat/diffusely infiltrative (type IV) according to the Borrmann classification [20].

The amplification or overexpression of HER2 is the main driver of tumorigenesis and is evident in 10-30% of gastric cancers, helping to serve as a predictive and prognostic biomarker [17,21]. This is supported by a recent study conducted in 2011 that showed patients with HER2-positive cancer of all stages had a poorer prognosis compared to HER2-negative patients [22]. Despite this, there have been conflicting results that highlight that HER2 may not be associated with prognostic outcomes in gastric cancer [23]. As such, the utility of HER2 as a prognostic marker is limited. The role of HER2 as a potential target for targeted therapy has shown promising results, and trials are currently underway. Trastuzumab is a monoclonal antibody that has shown promising results in HER2-positive patients. It is thought to work by inhibiting HER2-downstream signalling pathways. One study showed the addition of trastuzumab to chemotherapy compared to chemotherapy alone increased overall survival in patients with gastric cancer [24]. In addition, this links well with another trial that revealed trastuzumab in combination with the topoisomerase inhibitor deruxtecan increased overall survival compared to the chemotherapy agents irinotecan and paclitaxel [25].

Despite this, intratumor heterogeneity plays a key role in resistance and failure to respond to trastuzumab. To help overcome resistance, new therapeutic strategies have been developed that target inflammatory and immune checkpoint inhibition [26]. This is seen with pembrolizumab, which is a programmed death-1 (PD-1) inhibitor preventing the coupling of PD-1 and programmed death-ligand 1 (PD-L1) found on T cells and tumour cells, respectively. As the inhibitory interaction of PD-1/PD-L1 is prevented, the effect of the cytotoxic T-cell antitumour response is enhanced in the tumour microenvironment [27]. Indeed, a phase III clinical trial demonstrated that the addition of pembrolizumab to trastuzumab and chemotherapy markedly increased the tumour objective response rate in HER2-positive gastric cancer [28]. As such, the addition of immunotherapy to targeted therapy in combination with standard chemotherapy offers further potential avenues to help tackle the high tumorigenicity and acquired resistance evident in gastric cancer. The reason for HER2-positive gastric tumours failing to respond to trastuzumab may involve complex crosstalk between signalling pathways. The addition of inhibitory treatments that mediate cell signalling may help overcome trastuzumab resistance. The mammalian target of the rapamycin (mTOR) signalling pathway regulates cell growth, apoptosis, and autophagy [29]. It is also highly expressed in gastric cancer, and blocking this pathway may inhibit tumour proliferation [30]. A recent trial showed the addition of mTOR inhibitors to HER2-transtuzumab-resistant gastric cancer cell lines displayed considerable cell inhibition [31]. This indicates the addition of mTOR inhibitors may present a new therapeutic approach to tackling trastuzumab resistance in HER2-positive gastric cancer. Another key novel anti-HER2 therapy is seen with pertuzumab. Pertuzumab prevents the heterodimerisation of HER2 with other family receptors, such as human epidermal growth factor receptor-3 (HER3), thus avoiding a potent pro-oncogenic heterocomplex [32]. This abrogates the transduction of signalling pathways involved in tumorigenesis. This was seen in a recent trial that showed the addition of pertuzumab to trastuzumab and chemotherapy increased overall survival and tumour objective survival rates [33]. This further demonstrates alternative avenues to target HER2-positive gastric tumours.

Human epidermal growth factor receptor-1 (HER1), unlike HER2, is a ligand-dependent tyrosine kinase receptor. The binding of a specific ligand induces conformational change and subsequent dimerisation, initiating downstream signalling pathways that regulate cell growth, differentiation, migration, and survival [34]. The overexpression and amplification of HER1 in patients with gastric cancer, as determined by immunohistochemistry and FISH, is correlated with a poor prognosis [35]. However, a meta-analysis showed conflicting results regarding the prognostic significance of HER1 in gastric cancer and its potential role as a biomarker [36]. The use of directed therapy against HER1-status gastric cancer has been disappointing. Cetuximab is a monoclonal antibody that acts as a competitive inhibitor, preventing the binding of ligands to HER1. The intended aim is to inhibit the growth and survival of HER1-positive gastric tumour cells. However, a randomised trial revealed the supplementation of cetuximab with chemotherapy therapy agents capecitabine-cisplatin had no benefit to progression-free survival in contrast to chemotherapy alone [37]. Panitumumab is another monoclonal antibody HER1 antagonist that, like cetuximab, has demonstrated no activity in HER1-status gastric cancer [38]. Overall, there is limited evidence available on the prognostic value of HER1 as a biomarker and the relevance of anti-HER1-directed treatment in gastric cancer.

One further biomarker that is also a member of the receptor tyrosine kinase family and is expressed in a significant proportion of gastric cancer biopsies is c-mesenchymal epithelial transition factor (c-MET) [39]. The respective ligands, hepatocyte growth factor (HGF) or scatter factor (SF), bind to the receptor and activate a cascade of signalling mechanisms that regulate cell proliferation, motility, and survival [39]. The overexpression or amplification of c-MET is evident in gastric cancer, which helps to promote tumour cell growth, angiogenesis, metastasis, and survival [40]. The identification of c-MET as a molecular biomarker in gastric cancer has been correlated with a poor prognosis [41-43]. Novel targeted therapy against c-MET and corresponding ligands has shown promising results. Rilotumumab, a monoclonal antibody, prevents the coupling of HGF or SF to the c-MET receptor, thus disrupting signalling pathways that promote tumorigenesis. It was shown in a trial that the addition of rilotumumab to a chemotherapy regimen helped to advance progression-free survival [44]. This was also seen in another study whereby the addition of rilotumumab to chemotherapy resulted in greater inhibition of tumour cell growth [45]. Overall, results are encouraging thus far, and further studies are warranted to fully elucidate the role of c-MET and targeted therapy in gastric cancer.

Angiogenesis is one of the hallmarks of cancer and is a key rate-limiting step in tumorigenesis. One main driving proangiogenic factor is vascular endothelial growth factor (VEGF) and its cognate tyrosine kinase receptor, vascular endothelial growth factor receptor-2 (VEGFR2). Tumour cells release VEGF in response to hypoxia [46]. The canonical activation involves VEGF binding to VEGFR2 and subsequent dimerisation and autophosphorylation of tyrosine residues. This activates intracellular signalling pathways integral to angiogenesis, which helps facilitate nutrition and oxygen for cell growth, migration, and survival [47]. The importance of VEGF and VEGFR2 as prognostic biomarkers in gastric cancer has been investigated in several studies. A previous study suggests the upregulation and presence of VEGF in gastric tumours were associated with shorter survival rates compared to VEGF-negative gastric tumours [48]. Further studies indicate the expression of VEGFR2 in gastric cancer is associated with poor survival and can help stratify patients who are at high risk of poor prognosis [49-52]. Consequently, the therapeutic implications of targeted therapy against both VEGF and VEGFR2 may be significant in the management of gastric cancer.

There have been recent advancements in targeting the ligand VEGF and its cognate receptor, VEGFR2. One trial showed the use of an anti-VEGF monoclonal antibody, bevacizumab, as monotherapy inhibited tumour growth and metastasis [53]. The synergistic effects of adding bevacizumab to chemotherapy resulted in greater anti-tumour activity [53]. In gastric cancer, one study showed the addition of bevacizumab to chemotherapy improved response rate, time to progression, and overall survival in patients [54]. Furthermore, another study showed that the use of bevacizumab with chemotherapy improved progression-free survival [55]. The supplementation of bevacizumab with chemotherapy was also shown to improve the overall response rate and progression-free survival in patients with advanced gastric cancer [56]. This demonstrates the significant role of anti-VEGF targeted therapy in treating patients with VEGF-positive gastric cancer. The role of targeted therapy against VEGFR2 has also shown success in several studies. Ramucirumab, a monoclonal antibody, is an anti-angiogenic agent with a high degree of specificity and affinity to the VEGF binding domain on VEGFR2 [57]. This prevents the coupling of VEGF and VEGFR2, thus abrogating downstream signalling that mediates angiogenesis [57]. It has shown promising anti-tumour activity in patients with VEGFR2-positive cancer [58]. In gastric cancer, a randomised controlled trial showed the use of ramucirumab with the best supportive care increased progression-free and overall survival in metastatic gastric cancer [59]. This was also evident in a further study that showed the use of ramucirumab as monotherapy prolonged survival compared to a placebo in gastric cancer [60]. Ramucirumab combined with chemotherapy has also been shown to extend survival in advanced gastric cancer [61]. These findings are positive and help to support the potential role of targeted therapy against VEGFR2 signalling in gastric cancer.

hERG1 as a biomarker and the therapeutic implications in gastric cancer

There is variation in the efficacy of targeted therapy seen in patients with gastric cancer. This is primarily due to resistance through adaptive bypass mechanisms or the disruption of negative feedback loops. This enables tumour cells to evade targeted therapy. A better understanding of tumourigenesis has helped to better appreciate the importance of the tumour microenvironment in neoplastic progression. The microenvironment undergoes remodelling and is essential for the survival, function, and metastasis of tumour cells. Novel therapeutic agents can be developed to help overcome resistance by targeting the bidirectional interaction between tumour cells and the complex tumour microenvironment. One approach to disrupting signalling and crosstalk is by directing therapy towards ion channels. Indeed, ion channels are misexpressed in tumours [62]. An example is the hERG1, which is modulated by a hypoxic niche and, through the activity of hypoxia-inducible factor-1α (HIF-1α), plays a central role in angiogenesis and chemoresistance [63].

The expression of hERG1 is found in several tissues. These include smooth muscle, neural, and cardiac myocytes, where it has an integral physiological role in mediating action potential repolarisation [64]. The hERG1 protein is not normally prevalent in the mucosa of the stomach [65]. In the event of hypoxia, caused by rapid tumour cell proliferation exceeding neovascularisation, aberrant hERG1 ion channels are upregulated. Interestingly, one study found hERG1 expression to be a marker of the early stages of gastric carcinogenesis [65]. The overexpression of hERG1 is evident in gastric cancer, where it regulates cell proliferation and apoptosis [66]. This voltage-dependent potassium channel mediates the release of VEGF and has been shown to be pivotal in different processes such as cell growth, apoptosis, migration, and regulating angiogenesis. This crosstalk interaction between overexpressed hERG1 and VEGF release is evident in gastric cancer and serves as a potential novel biomarker and therapeutic target [67].

The aberrant expression of hERG1 identified by immunohistochemistry has been shown to be paramount in high-grade gastric dysplasia - a precancerous lesion of intestinal gastric cancer. This was shown to reduce overall survival and thus offers a potential role as a poor prognostic biomarker of disease progression [68]. In addition, a further study demonstrates that hERG1 expression in gastric cancer is correlated with tumour progression, invasive phenotypes, and poor survival [69]. The invasiveness, proliferation, and tumorigenicity of gastric cancer cells expressing hERG1 were also seen in another study [66]. This further highlights the significance of hERG1 as a predictive, diagnostic, and prognostic biomarker in gastric cancer.

There have been several developments in the potential implications of the hERG1 ion channel as a therapeutic target. One study indicates that inhibition and silencing of the hERG1 protein prevent the proliferation and invasiveness of gastric cancer cells [66]. Furthermore, tumour cells do not advance into the replication S phase of the cell cycle and instead are subjected to arrest and subsequent apoptosis [66]. A recent study demonstrated that the use of a hERG1 blocker reduced gastric tumour volume and angiogenesis with greater inhibitory effects when combined with bevacizumab [65]. The use of hERG1 was also shown to be significant in mediating the effects of apoptosis when used alongside the chemotherapy agent cisplatin in gastric cancer [70]. Despite this, care must be taken as hERG1 antagonism poses cardiotoxic side effects with increased risks for QT prolongation, arrhythmia, and sudden cardiac death [71]. Although, there have been efforts to develop selective hERG1 inhibitors that halt tumour progression while mitigating cardiotoxicity [72]. Overall, these findings suggest that the antagonism of hERG1 potentiates the inhibition of neoplastic progression. This offers a novel and personalised targeted therapy plan for patients with gastric cancer.

## Conclusions

In conclusion, there have been recent therapeutic advancements to help manage the high degree of heterogeneity and acquired resistance seen in gastric cancer. Several current biomarkers have been shown to have predictive, diagnostic, and prognostic value. This enables the stratification of high-risk individuals and allows a personalised, targeted therapeutic approach to treating gastric cancer patients. An emerging biomarker that has demonstrated predictive, diagnostic, and prognostic significance is hERG1. The antagonism of hERG1 has been shown to have synergistic effects with standard chemotherapy, further potentiating tumour regression. In the future, the implementation of hERG1 as a molecular biomarker and its use in targeted therapy will offer a new approach to the personalised management of gastric cancer.
